# Internet addiction and mental health: a cross-sectional study and mediation analysis in medical students with a psychiatric major

**DOI:** 10.3389/fpsyt.2025.1625824

**Published:** 2025-07-25

**Authors:** Ying Lu, Yue Lu, Yu Tian, Yu Gan, Xiaolu Chen, Xiao Li

**Affiliations:** ^1^ Department of Psychiatry, The First Affiliated Hospital of Chongqing Medical University, Chongqing, China; ^2^ Department of the First Clinical Medicine, Chongqing Medical University, Chongqing, China; ^3^ Sleep & Psychosomatic Medical Center-ward, Dazu Hospital of Chongqing Medical University, Chongqing, China; ^4^ Department of Psychiatry, Chongqing Yongchuan Mental Health Center, Chongqing, China; ^5^ Department of Psychiatry, Chongqing Eleventh People’s Hospital, Chongqing, China; ^6^ Department of Psychiatry, Key Laboratory of Major Brain Disease and Aging Research (Ministry of Education), The First Affiliated Hospital of Chongqing Medical University, Chongqing, China

**Keywords:** internet addiction (IA), medical students, depression, anxiety, impulsivity, social support

## Abstract

**Background:**

Internet Addiction (IA) has become a significant public health issue, particularly among medical students. However, research on IA within medical students with a psychiatric major, who receive specialized mental health training, remains limited. This study aimed to examine the prevalence of IA and its associations with mental health variables (depression, anxiety, impulsivity) and social factors among medical students with a psychiatric major, while exploring the mediating role of mental health variables in the relationship between academic year and IA.

**Methods:**

A cross-sectional survey was conducted with 390 medical students with a psychiatric major at Chongqing Medical University from February to May 2021. Participants completed validated instruments, including Young’s Internet Addiction Test (Y-IAT), Patient Health Questionnaire-9 (PHQ-9), Generalized Anxiety Disorder-7 (GAD-7), and Barratt Impulsiveness Scale (BIS-11). Demographic and social factors were analyzed using Mann-Whitney U tests, chi-square test, correlation analyses. Mediation analyses were conducted using the SPSS PROCESS macro (Model 4).

**Results:**

The average Y-IAT score was 37.57 ± 13.80, with 17.7% meeting the criteria for IA (Y-IAT ≥50). Additionally, participants reported low levels of depressive (PHQ-9 = 2.04 ± 3.01) and anxiety symptoms (GAD-7 = 1.26 ± 2.58). IA was significantly associated with increased impulsivity (r = 0.534, p < 0.001), depression (r = 0.399, p < 0.001), anxiety (r = 0.347, p < 0.001), academic stress (r = 0.239, p < 0.001), poor peer relationships (r = 0.199, p < 0.001), and peer bullying experiences (r = 0.137, p < 0.05). A higher academic year was associated with a reduction in IA, partly mediated by decreased impulsivity (B = −0.7556, 36.60%) and depressive symptoms (B = −0.2640, 12.80%).

**Conclusion:**

Medical students with a psychiatric major showed a relatively lower prevalence of IA, depressive and anxiety symptoms. Higher academic years were associated with reduced IA via lower impulsivity and depressive symptoms, potentially due to enhanced psychological literacy. Additionally, poor peer and parental relationships, along with experiences of peer bullying, were linked to higher IA risk, highlighting the importance of stronger social support and early intervention. Future studies should explore targeted psychological and educational interventions to mitigate IA among students.

## 1 Introduction

With the widespread adoption of the internet, the number of global internet users has reached 5.66 billion, accounting for 67.9% of the global population (1). As the country with the largest number of internet users, China had 1.108 billion internet users by 2024, with an internet penetration rate of 78.6% (2). While moderate internet use can be beneficial, excessive or uncontrolled use may lead to adverse consequences, including the development of Internet Addiction(IA). In 1995, American psychiatrist I. Goldberg first introduced the concept of IA to describe pathological and compulsive online behavior (3). This term has several synonyms, such as “Pathological Internet Use,” “Problematic Internet Use,” or “Compulsive-Impulsive Internet Use Disorder.” However, there is no clear definition of this concept in the academic community, and these terms are often used interchangeably in the literature (4). In this paper, unless quoting directly, the term “IA” will be used consistently. Although Internet Gaming Disorder (IGD) is classified as a disorder in both DSM-5 and ICD-11, it is important to note that IA has not been recognized as a distinct disorder in either diagnostic manual (3). The American Psychiatric Association has stated that IA requires further research before it can be formally classified (5). Current limitations in IA research include the lack of standardized diagnostic criteria, insufficient studies conducted in clinical contexts, and limited neurobiological and genetic evidence (6). Therefore, it is necessary to conduct more standardized and clinically validated research to advance understanding of IA and its potential classification as a formal disorder.

IA has become an increasingly serious issue among medical students. A 2020 meta-analysis estimated the global prevalence of General Internet Addiction (GIA) at 7.02% (7). However, the prevalence among medical students is significantly higher. A 2018 meta-analysis reported a prevalence of 30.1% among medical students (8), and a 2024 study found a prevalence of 29% (3), nearly four times higher than that of the general population. Research has shown that IA is closely associated with various negative psychological and behavioral outcomes, particularly among medical students. It is known to be linked to depression (9–11), anxiety (9, 11), low self-esteem (11), poor academic performance (12, 13), poor sleep quality (12, 14, 15), insomnia (11), and impulsivity (16). Furthermore, a neuroimaging meta-analysis of MRI studies revealed that young adults and adolescents with IA and social media addiction exhibit abnormalities in brain function and resting-state activity compared to control groups, including functional impairments in reward system and executive function (17). Impaired executive function can directly affect decision-making abilities, which in turn poses a threat to patient safety. Therefore, the impact of IA on medical students, who are the future of the medical profession, is particularly concerning. Medical students not only face a higher risk of IA but also experience its negative effects on their psychological health and academic performance, which may further impact their future clinical practice and patient care.

Unlike in many other countries where medical students obtain a Bachelor of Medicine, Bachelor of Surgery (MBBS) or Doctor of Medicine (MD) degree before choosing psychiatry as a specialization during postgraduate residency, China has developed a unique medical education model to address the shortage of psychiatric professionals. Several medical universities in China, including Central South University, Capital Medical University, Nanjing Medical University, Guangzhou Medical University, Southern Medical University, and Chongqing Medical University, have established dedicated Bachelor’s programs in Psychiatry. This undergraduate Psychiatry program in China is structured similarly to the standard Clinical Medicine program. Students are admitted directly after graduating from high school through the National College Entrance Examination, following the same admission process as other undergraduate majors in China. The program spans five years, after which graduates must complete a three-year Standardized Residency Training Program and pass the Chinese Medical Licensing Examination in order to qualify as licensed physicians.

The overall curriculum is nearly identical to that of the Clinical Medicine program, with the key distinction being enhanced training in psychiatry and psychology, including additional coursework and clinical exposure. Therefore, graduates from the Psychiatry program are eligible to pursue not only psychiatry but also other medical specialties. In a separate study conducted by our team (18), students enrolled in the Psychiatry undergraduate program demonstrated more favorable attitudes toward psychiatry. This enhanced perception of psychiatry may influence their career intentions and increase the likelihood of choosing psychiatry as a future specialization.

Chongqing Medical University, established in 1956 and located in Chongqing, China, currently offers psychiatric programs leading to Bachelor’s, Master’s, and Doctoral degrees. Since 2016, the university has launched a Bachelor’s program in Psychiatry to train more psychiatric professionals, enrolling approximately 80 students per year. Compared to standard Clinical Medicine Programs in China, those enrolled in the Psychiatry program receive more comprehensive psychiatric coursework. Given this specialized training, we speculate that medical students with a psychiatric major may exhibit different patterns of IA and mental health outcomes compared to Clinical Medicine students due to their increased exposure to psychiatry-related coursework.

Currently, there is no research on IA and its relationship with mental health among medical students with a psychiatric major. We hypothesize that the prevalence of IA and mental health conditions among medical students with a psychiatric major may be better than that of Clinical Medicine students. Additionally, as students progress through their academic years, their mental health may improve, which could indirectly influence IA. This study aims to test this hypothesis and explore the correlation between IA, mental health, and social factors in this population.

## 2 Materials and methods

### 2.1 Participants and procedure

From February to May 2021, a total of 390 students from Chongqing Medical University, spanning from the 1st to the 5th year, participated in this study on a voluntary basis. The students completed an online questionnaire that included several scales. This study did not provide financial compensation or disclose participant identities. Ethical approval was obtained from the Human Research and Ethics Committee of the First Affiliated Hospital of Chongqing Medical University, and informed consent was obtained from all participants.

### 2.2 Measures

#### 2.2.1 Demographic variables

Participants completed a questionnaire that collected demographic and general information. The details included age, gender, academic year (from 1-5); relationship between parents (good/poor), relationship with parents (good/average), family economic status (good or average/poor); relationship with classmates (good/average); smoking history; alcohol consumption history; level of study pressure (high or average/low); bullying experience by classmates (yes/no); only child (yes/no); and the location of the family’s residence (urban/rural).

#### 2.2.2 Young’s Internet Addiction Test

The 20-item Young’s Internet Addiction Test (Y-IAT), developed by Young et al., is a modified version of Young’s Diagnostic Questionnaire for IA (19). It is a 20-item self-report questionnaire designed to measure an individual’s internet use. Each item is rated on a 5-point scale, ranging from 1 (very rarely) to 5 (very frequently), with a total score ranging from 20 to 100. Higher scores indicate a greater tendency toward IA, and an IAT score of ≥50 is considered indicative of IA in this study. The Chinese version of the Y-IAT was used in this study. The Cronbach’s alpha for the Y-IAT was 0.944.

#### 2.2.3 Barratt Impulsiveness Scale-11

The current 11th version of the Barratt Impulsiveness Scale (BIS-11) is a 30-item self-report instrument. It has been tested for reliability and validity across various populations in China (20). The BIS-11 consists of three factors that assess different aspects of impulsivity: non-planning impulsiveness, motor impulsiveness, and attentional impulsiveness. In the present study, the BIS-11 was administered in its validated Chinese version and the Cronbach’s alpha was 0.928.

#### 2.2.4 Patient health questionnaire-9

The PHQ-9 is a self-report questionnaire consisting of nine items to assess symptoms of depression (21). The questionnaire evaluates whether the symptoms have bothered the individual in the past two weeks. Each item is scored from 0 to 3, with the total score ranging from 0 to 27. Based on the summary score, the severity of depression can be assessed: scores of 5–9 indicate mild depression, 10–14 indicate moderate depression, 15–19 indicate moderately severe depression, and ≥ 20 indicates severe depression. The reliability and validity of the PHQ-9 have been tested and confirmed in China (22). In this study, a score of 5 or above represented the presence of depressive symptoms. The PHQ-9 was administered in Chinese and Cronbach’s alpha was 0.848 in this study.

#### 2.2.5 Generalized Anxiety Disorder-7

The Generalized Anxiety Disorder-7 (GAD-7) was developed by Spitzer et al. (23) as a seven-item self-rating scale designed to screen for generalized anxiety disorder (GAD) and assess its severity. Each item corresponds to a typical symptom of GAD, with responses based on the frequency of occurrence over the past two weeks: “Not at all” scores 0, “Several days” scores 1, “More than half the days” scores 2, and “Nearly every day” scores 3. The GAD-7 was translated into Chinese in 2010 and validated in a sample of outpatients at a general hospital (24). In this study, a score of 5 or higher indicated the presence of anxiety symptoms. In this study, the Chinese version of the GAD-7 was used and the Cronbach’s alpha for the GAD-7 was 0.908.

### 2.3 Statistical analysis

Before conducting statistical analyses, we assessed the normality of the data using Kolmogorov-Smirnov and Shapiro-Wilk tests. Given that most variables did not meet the assumption of normality, Chi-square tests and Mann-Whitney U tests were used to compare demographic and clinical characteristics between the IA and non-IA groups. To further explore the relationships between IA and other psychological factors, Pearson correlation analyses were conducted for Y-IAT scores and variables that showed significant differences in group comparisons. Additionally, we examined the correlations between academic year and these psychological variables to investigate whether academic year plays a potential role in influencing IA through these factors. To test whether depressive symptoms (PHQ-9), anxiety symptoms (GAD-7), and impulsivity (BIS-11) mediate the relationship between academic year and IA, we performed a series of mediation analyses using Hayes’ SPSS Process Macro (version 4.2, Model 4) (25). A bootstrapping method with 5000 resamples was applied to estimate indirect effects, with a 95% confidence interval for the analysis. All statistical analyses were conducted using SPSS version 29.0, and a two-tailed p-value < 0.05 was considered statistically significant.

## 3 Result

### 3.1 Demographic characteristics

In the total sample, 69.2% of participants were female (n = 290), with a mean age of 21.31 ± 1.26 years and an average Y-IAT score of 37.57 ± 13.80. A total of 17.7% of participants (n = 69) met the criteria for IA. **Table 1** presents a comparison of demographic characteristics between the IA group (n = 69) and the non-IA group (n = 321). The results indicate that individuals in the IA group were significantly younger (p = 0.025), experienced higher academic stress (p < 0.001), had poorer relationships with their parents (p = 0.011) and peers (p = 0.001), and were more frequently subjected to peer bullying (p < 0.001) compared to those in the non-IA group. No significant differences were found between the two groups in terms of gender, smoking history, alcohol consumption history, parental marital status, family economic status, only-child status, or family residence location.

**Table 1:** Demographic characteristics of IA and non-IA groups.

Characteristics	IA N=69	Non-IA N=321	U/χ²	p
Age (Median, IQR)	21 (2)	21 (3)	9205.500*	0.025
Gender (female, %)	51 (73.9%)	219 (68.2%)	0.863	0.353
Smoking History	2 (2.9%)	23 (7.2%)	2.056	0.152
Alcohol History	34 (49.3%)	142 (44.2%)	0.582	0.258
Academic Stress	37 (53.6%)	67 (20.9%)	31.151**	<0.001
Poor relationship between parents	16 (23.2%)	68 (21.2%)	0.135	0.713
Poor Family Economy	8 (13.0%)	42 (13.1%)	0.000	0.993
Poor Relationship with Parents	21 (30.4%)	55 (17.1%)	6.404*	0.011
Poor Peer Relationship	28 (40.6%)	71 (22.1%)	10.219**	0.001
Peer Bullying Experience	10 (14.5%)	8 (2.5%)	13.908**	<0.001
Only Child	28 (40.6%)	153 (47.7%)	1.146	0.284
Residence (rural area)	15 (21.7%)	73 (22.7%)	0.033	0.857

* p < 0.05, ** p < 0.001. IA, Internet Addiction; IQR, Interquartile Range.

### 3.2 Clinical characteristics

In the total sample, 16.2% of participants (N = 63) reported depressive symptoms (PHQ-9 ≥ 5), with an average score of 2.04 ± 3.01, while 9.2% (N = 36) reported symptoms of anxiety (GAD-7 ≥ 5), with an average score of 1.26 ± 2.58. The results of the Mann-Whitney U test indicated significant differences between the IA and Non-IA groups in impulsivity and depressive symptoms. Specifically, the IA group exhibited significantly higher BIS scores (U = 5176.0, p < 0.001) and PHQ-9 scores (U = 6445.0, p < 0.001). However, no significant difference was found in GAD-7 scores (U = 7379.5, p = 0.085). The results are shown in **Table 2**.

**Table 2:** Comparison of depression, anxiety, and impulsivity between IA and non-IA groups.

Scales	IA (N=69) median (IQR)	Non-IA (N=321) median (IQR)	U	p
BIS-11	61 (22)	46 (21.5)	5176.000**	<0.001
GAD-7	1 (2)	0 (1)	7379.500	0.085
PHQ-9	3 (5.5)	1 (2)	6445.000**	<0.001

**p < 0.001. IA, Internet Addiction; IQR, Interquartile Range; BIS, Barratt Impulsiveness Scale-11; GAD-7, Generalized Anxiety Disorder-7; PHQ-9, Patient Health Questionnaire-9.

### 3.3 Correlation analysis

**Table 3** shows the results of the correlation analysis. The correlation analysis revealed that Y-IAT scores were significantly positively correlated with BIS scores (r = 0.534, p < 0.001), GAD-7 scores (r = 0.347, p < 0.001), PHQ-9 scores (r = 0.399, p < 0.001), academic stress (r = 0.239, p < 0.001), poor relationship with parents (r = 0.104, p < 0.05), poor peer relationship (r = 0.199, p < 0.001), and peer bullying experiences (r = 0.137, p < 0.05). Conversely, Academic year was significantly negatively correlated with Y-IAT scores (r = -0.238, p < 0.001), BIS scores (r = -0.174, p < 0.001), GAD-7 scores (r = -0.187, p < 0.001), and PHQ-9 scores (r = -0.131, p < 0.001). Additionally, a gender-stratified analysis showed that the correlation between BIS and Y-IAT scores was stronger in male students (r = 0.645, p < 0.001) than in female students (r = 0.469, p < 0.001).

**Table 3:** Correlation analysis of Y-IAT scores with clinical and demographic variables.

	Y-IAT	BIS	Academic Year	GAD-7	PHQ-9	Academic stress	Poor relationship with parents	Poor peer relationship	Peer bullying experience
Y-IAT	1								
BIS-11	0.534**	1							
Academic year	-0.238**	-0.174**	1						
GAD-7	0.347**	0.416**	-0.187**	1					
PHQ-9	0.399**	0.402**	-0.131**	0.654**	1				
Academic Stress	0.239**	0.236**	0.086	0.281**	0.213**	1			
Poor Relationship with Parents	0.104*	0.073	0.047	0.188**	0.206**	0.113*	1		
Poor Peer Relationship	0.199**	0.139*	-0.005	0.189**	0.206**	0.115*	0.368**	1	
Peer Bullying Experience	0.137*	0.165**	-0.064	0.137**	0.129*	0.033	0.077	0.012	1

*p < 0.05, **p < 0.001. IA, Internet Addiction; Y-IAT, Young’s Internet Addiction Test; BIS, Barratt Impulsiveness Scale-11; GAD-7, Generalized Anxiety Disorder-7; PHQ-9, Patient Health Questionnaire-9.

### 3.4 Mediation analysis

**Figure 1:**
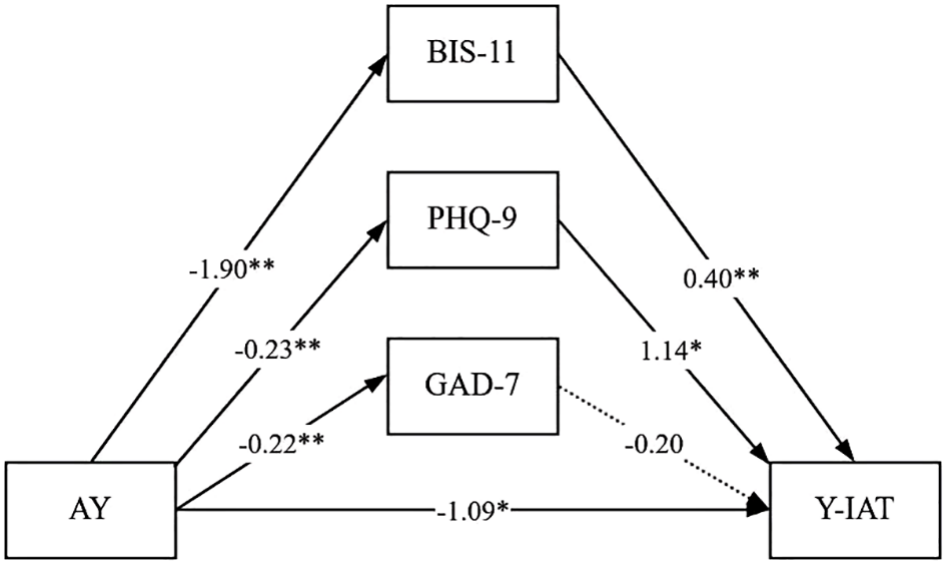
Mediation pathways from academic year to IA via psychological variables. Proposed mediation model illustrating the indirect effects of academic year (AY) on IA (Y-IAT) through impulsivity (BIS-11), depression (PHQ-9), and anxiety (GAD-7). Path coefficients are standardized (β values).

As shown in the mediation model (**Figure 1**) and the effect decomposition results (**Table 4**), academic year exhibited a significant negative total effect on IA (Y-IAT) (B = -2.06, p < 0.001), indicating that students in higher academic years had significantly lower levels of IA. After accounting for the mediating variables, the direct negative effect of academic year on IA remained significant (B = -1.09, p = 0.0087). Academic year indirectly reduced IA by lowering impulsivity (BIS-11) (B = -0.76, bootstrap 95% CI [-1.2715, -0.3143]), contributing 36.60% of the total effect. It also indirectly reduced the risk of IA by alleviating depressive symptoms (PHQ-9) (B = -0.26, bootstrap 95% CI [-0.5542, -0.0396]), contributing 12.80%. However, the mediating effect of anxiety symptoms (GAD-7) was not significant (B = 0.04, bootstrap 95% CI [-0.1368, 0.2725]), and the direct path from GAD-7 to Y-IAT was also not significant (**Figure 1**), indicating that anxiety did not serve as an effective mediator in this model.

**Table 4:** Effect decomposition of academic year on IA.

Effect	Path	B	SE	t	95% CI	
					Lower	Upper
Total Effect	AY→Y-IAT	-2.06	0.48	-4.28**	-3.0116	-1.1167
Direct Effect	AY→Y-IAT	-1.09	0.41	-2.64*	-1.8990	-0.2767
Indirect Effect	AY→BIS-11→Y-IAT	-0.76	0.24		-1.2715	-0.3143
	AY→PHQ-9→Y-IAT	-0.26	0.13		-0.5542	-0.0396
	AY→GAD-7→Y-IAT	0.04	0.10		-0.1368	0.2725

*p < 0.05, **p < 0.001. AY, Academic Year; Y-IAT, Young’s Internet Addiction Test; BIS-11, Barratt Impulsiveness Scale-11; GAD-7, Generalized Anxiety Disorder-7; PHQ-9, Patient Health Questionnaire-9.

## 4 Discussion

### 4.1 Mental health status and prevalence of IA among medical students with a psychiatric major

This study found that 16.2% (N=63) of medical students with a psychiatric major exhibited depressive symptoms (PHQ-9 ≥ 5), with an average score of 2.04 ± 3.01. Meanwhile, 9.2% (N=36) reported anxiety symptoms (GAD-7 ≥ 5), with an average score of 1.26 ± 2.58. These rates are significantly lower than those reported among Greek undergraduate medical students, who had higher mean anxiety (9.04 ± 5.66) and depressive (9.36 ± 6.15) scores based on the same criteria (26). Similarly, during the COVID-19 pandemic, the anxiety rate among our group was lower than that observed in medical undergraduates at that time (24.9%) (27), and also lower than the post-pandemic rates of anxiety (37.8%) and depression (39.3%) observed among medical undergraduates in China (28). These findings suggest that medical students with a psychiatric major may have better mental health compared to both the general undergraduate and medical student populations.

Regarding the prevalence of IA, the rate in our cohort (17.7%) was lower than the 24.3% reported in a meta-analysis of Asian university students (4) and the 29% found in a meta-analysis of global medical students (3). However, it is important to note that differences in measurement tools and scoring criteria across meta-analyses can lead to substantial variations in prevalence estimates (29). When comparing studies that also used the Y-IAT≥50 criterion, our prevalence rate is higher than the 9.2% reported in a 2019 cross-sectional study of Chinese medical students (30). Given that our data were collected in 2021, this difference may be related to the widespread implementation of isolation measures and online education during the COVID-19 pandemic. A study from the general population in China showed that time spent on internet use significantly increased during the pandemic, with almost half of the participants reporting a greater severity of IA (31). An Italian study found that COVID-19 was associated with depression, anxiety, social isolation, and problematic Internet use (32). When compared to other studies using the Y-IAT≥50 criterion during the COVID-19 pandemic, our prevalence is lower than the 27.7% reported in a multinational cross-sectional survey of medical students from other countries (15), and it is also lower than the 28.4% reported in a 2021 study of Chinese university students (33). According to a meta-analysis, the rate of IA among medical students is five times higher than that in the general population (8). Studies using the same criterion indicate that the rate of IA in our cohort is lower than that in most medical student populations and even lower than that of the general university student population in China.

The proportion of medical students with a psychiatric students with depression and anxiety symptoms, as well as the prevalence of IA, is lower than that observed in existing data from medical and general university student populations. This may be attributed to the unique characteristics of psychiatric students, whose curriculum includes substantial mental health-related coursework, which may contribute to enhanced psychological well-being. For instance, according to the curriculum plan for the Class of 2020 psychiatric students at Chongqing Medical University (who were first-year students when the questionnaire was administered), they are required to complete 60.5 credits in psychology- or psychiatry-related courses (one credit equals 8 hours of coursework), including courses such as General Psychology, Medical Psychology, Psychiatry, Psychosomatic and Behavioral Medicine, Psychiatric Pharmacology, and Community and Judicial Psychiatry. This translates to a total of 484 hours in these areas throughout their five-year undergraduate program. One study has shown that the primary factor influencing differences in mental health literacy scores is participation in clinical psychology courses, with psychology as a major being the second most influential factor (34). Similarly, other studies have also found that psychology majors are associated with higher mental health literacy (35–37). Another study reported that nursing students who took mental health courses were more likely to seek professional psychological help compared to before (38).

The concept of “mental health literacy,” coined by Anthony F. Jorm in 1997, refers to the knowledge and beliefs about mental disorders that facilitate their recognition, management, or prevention (39). Psychology-related courses may enhance students’ psychological literacy, which, in turn, could promote the early identification and intervention of mental health issues. This may help explain the lower rates of depression, anxiety, and IA observed in our cohort. These findings suggest that strengthening mental health education among university students, particularly medical students, may be an effective strategy to improve psychological literacy and address mental health challenges and IA. In addition, it may be beneficial for psychiatrists to provide psycho-education to their patients, as improving patients’ mental health literacy may contribute to symptom reduction. Further research is warranted to explore the relationship between psychology-related coursework and mental health outcomes, including IA.

### 4.2 The relationship between IA and depression, anxiety, and impulsivity in psychiatric students

#### 4.2.1 IA and mental health

This study found a significant positive correlation between IA (measured by Y-IAT scores) and anxiety symptoms (GAD-7 scores) as well as depressive symptoms (PHQ-9 scores) among medical students with a psychiatric major. A meta-analysis examining the association between IA and mental disorders, which included 23 studies (N=21,582), confirmed a moderate correlation between IA and both anxiety and depression (40). This phenomenon may be explained by the tendency of individuals with mental health issues to use the internet as a coping mechanism for negative emotions. As one study stated, the internet can serve as a platform to expand social networks, provide individuals with more meaningful relationships, boost self-esteem, improve social skills, and offer opportunities for social support (41).

However, excessive internet use may lead to IA, which can result in a range of negative consequences. These negative outcomes, in turn, may contribute to the development or exacerbation of depressive and anxiety symptoms. For example, excessive internet use has been associated with increased daytime sleepiness (42), reduced days of vigorous physical activity (43), decreased sleep quality (15) and fewer offline social activities. These factors may contribute to the development of depression and anxiety symptoms. IA is clearly closely related to mental health issues, but its causal relationship remains complex. Future research should employ longitudinal study designs to further explore the relationship between IA and mental disorders. Additionally, when treating IA, interventions should address not only IA itself but also the co-existing mental health issues.

#### 4.2.2 Impulsivity and IA

This study found a significant positive correlation between impulsivity (BIS-11 scores) and IA (Y-IAT scores) among psychiatric students. The relationship between impulsivity and IA has been reported in several studies (16, 44, 45). Our study further revealed that impulsivity was significantly correlated with IA in both male and female participants, with the correlation coefficient being slightly higher in males (r=0.645) than in females (r=0.469). This finding is consistent with a study conducted on medical students in northern Iran (16). A meta-analysis also found a positive correlation between impulsivity and IA, unaffected by gender (46). Overall, in line with previous studies, our research supports the view that IA should be considered an impulse control disorder, and that the relationship between impulsivity and IA is independent of gender.

A cross-diagnostic analysis found that motor impulsivity was closely related to symptoms associated with depression and anxiety (47). Correlation analysis in our study also revealed a significant positive correlation between impulsivity and both depression and anxiety. Additionally, in our study, impulsivity was also significantly associated with academic pressure, poor peer relationships, and experiences of peer bullying. A study on peer victimization, deviant peer affiliation, and impulsivity found that impulsivity moderated the direct association (peer victimization → problem behaviors) and the second stage of the indirect path (deviant peer affiliation → problem behaviors) (48). Future research should further explore the mechanisms linking impulsivity and these social-psychological factors in IA or other problem behaviors.

#### 4.2.3 Mediating role of academic year

This study found a negative correlation between academic year and depression, anxiety, and impulsivity. Further mediation analysis revealed that students in higher academic years had significantly lower IA levels, as measured by Y-IAT scores. This effect was partially mediated by reductions in impulsivity (BIS-11 scores) and depression (PHQ-9 scores). Additionally, academic year demonstrated a significant direct effect on Internet addiction (IA). However, in our mediation model, anxiety—as measured by the GAD-7—did not exhibit a significant mediating effect. Beyond the possible reduced statistical power due to low sample anxiety levels (M = 1.26, SD = 2.58), the inherently weak unique association between anxiety and IA appears critical. Correlation analyses confirmed an anxiety-IA link, yet its strength was markedly weaker than that of depression/impulsivity with IA. This suggests anxiety may not be a core emotional driver of IA. Given the high comorbidity between anxiety and depression, the observed anxiety-IA correlation likely stems from their shared variance with depression rather than anxiety’s independent effect. Particularly in our low-anxiety sample, anxiety’s potential contribution to IA may be further attenuated, explaining its non-significant mediation. An additional consideration is whether anxiety’s impact on IA requires specific contexts. For instance, intense academic pressure might trigger academic anxiety, which could directly promote escapist internet use and IA—a relationship potentially missed by generalized anxiety measures (e.g., GAD-7) in low-stress settings. Thus, future research should refine anxiety assessment by employing context-specific scales (e.g., academic anxiety instruments) to capture stressor-driven anxiety subtypes, while testing moderating effects through examining whether factors like academic pressure strengthen the anxiety-IA link. Such designs would better delineate the boundary conditions of anxiety’s role in IA.

At Chongqing Medical University, the first psychology-related course, General Psychology, is typically offered in the second semester of the sophomore year. This is followed by courses in psychiatry and Psychosomatic and Behavioral Medicine, typically scheduled for the second semester of the junior year, with other courses spread across the first and second semesters of the senior year. Therefore, the results of our mediation analysis may be explained by the increase in psychology- or psychiatry-related courses as students progress through their studies, which enhances their psychological literacy. Previous research has highlighted that participation in psychology courses is associated with higher psychological literacy (34), which, in turn, may contribute to reductions in anxiety, depression and impulsivity symptoms—psychological factors related to IA. Accordingly, in the mediation model, academic year indirectly reduced IAT scores by decreasing PHQ-9 and BIS-11 scores.

Another possible explanation is that this improvement may be related to challenges that first-year medical students compared to their more experienced peers. A qualitative study based on individual interviews indicated that first-year medical students encounter numerous challenges, including adapting to university life and transitioning into the role of medical student and future doctor (49). The study identified six major challenges for first-year medical students: wrong degree choice, mental health problems, acute crisis, at capacity, slow starter and family rock. These factors may contribute to heightened anxiety and depression, prompting students to seek solace through excessive internet use, which in turn increases their risk of developing IA. Additionally, since our sample consists of Chinese students, who have just completed the highly competitive college entrance examination, they have transitioned from strict school and parental control to managing their internet use independently. This sudden shift may lead to difficulties in self-regulation, increasing the likelihood of IA and further reducing social interactions, which in turn may exacerbate anxiety and depression symptoms.

A previously cited study also pointed out that students in their fourth year or beyond generally scored higher on psychological literacy tests than students in the first, second, or third year (34). The researchers suggested that this could be attributed to the accumulation of academic knowledge, life experience, and increased familiarity with the university environment, which facilitates access to mental health services and, consequently, improves psychological literacy. Therefore, as students advance in academic year, the combination of increased psychological literacy from psychology courses, professional knowledge accumulation, enriched life experience, and greater familiarity with mental health resources may contribute to reductions in depression, anxiety, impulsivity, and IA. Future research should further explore whether the beneficial effects of higher academic years on IA and mental health are specific to medical students with a psychiatric major or extend to the broader student population.

### 4.3 IA and social support among medical students with a psychiatric major

Our study found that Y-IAT scores were significantly correlated with poor relationships with parents, poor peer relationships, and peer bullying experiences. Consistent with previous research, social support has been shown to be negatively associated with IA (50–53). Specifically, regarding family dynamics, studies have indicated that poor family atmosphere and lack of love from parents were predictors of problematic internet use among college students (54). Additionally, poor parental attachment increases screen time usage and is associated with internet abuse in adolescents (55, 56). Adolescents with IA often experience insufficient parental involvement, supervision, and emotional availability, leading to lower levels of perceived social support (57). Moderately addicted young people are more likely to report poorer family emotional involvement and higher levels of impulsivity and depression (58). These findings align with our conclusion that individuals with poor relationships with parents are more likely to develop IA. On the one hand, individuals from dysfunctional family relationships may experience a lack of parental supervision and care, making them more prone to uncontrolled internet use or seeking psychological compensation online (59). On the other hand, poor family functioning is associated with psychological difficulties, such as depression and impulsivity, which further increase the risk of IA. A mediation study indicated that family function is negatively correlated with IA, with emotional distress and loneliness acting as mediators (60).

Consistent with a study on Chinese adolescent populations (61), student-student relationship has been confirmed as a protective factor against smartphone addiction. Our study further extends this finding by revealing a positive correlation between poor peer relationships and IA. However, an Egyptian study of medical students found that most students with IA (92%) had good peer relationships (p < 0.01) (10). One possible explanation for this discrepancy is that while poor peer relationships may drive individuals to excessive internet use for emotional compensation, good peer relationships do not necessarily prevent IA. The internet provides opportunities for individuals to maintain and expand their social connections, potentially increasing screen time regardless of their real-life social situation. One study suggests that online communication offers adolescents various opportunities, such as enhancing self-esteem, building relationships, improving friendship quality, and exploring sexual identity (62). Another study from China showed that online communication is positively correlated with adolescents’ subjective well-being (63). Thus, even individuals with strong offline social connections may still rely on internet interactions, increasing their risk of developing IA. Additionally, the differences in research outcomes may be influenced by the cultural contexts of different countries, and further research is needed to explore and understand this phenomenon.

Regarding the correlation between peer bullying experiences and IA, numerous studies have reported this association in adolescent populations (64–68). To the best of our knowledge, our study is the first to investigate this relationship in a university student population. Previous research suggests that depressive symptoms and anxiety symptoms mediate the association between peer victimization and IA (66), highlighting the importance of addressing these psychological factors in intervention efforts. A meta-analysis on the effectiveness of school-based anti-cyberbullying programs found that these programs effectively reduced the persistence of cyberbullying and the number of cyberbullying victims (69). Therefore, when assessing IA in adolescents or university students, it is crucial to consider their history of peer bullying. Implementing targeted interventions, such as psychological support services or school-based anti-bullying programs, could help mitigate mental health risks and reduce the likelihood of IA.

### 4.4 IA and other social factors among medical students with a psychiatric major

Our study found no significant correlation between IA and gender, being an only child, parental relationships, family economic status, or urban/rural background among medical students with a psychiatric major. These results contrast with some previous studies. For example, some studies have found that males are more likely to develop IA (10, 70, 71). However, other studies, including ours, have concluded that IA is not associated with gender (58). Besides, a study in nine European countries has suggested that women are more likely to develop IA than men (72). Further research indicates that women are more likely to become addicted to social media and smartphones, while men are more prone to addiction to online gaming (73, 74).

A study has identified being an only child, twins and having a rural background as predictive factors for internet gaming addiction among Chinese adolescents (74), but our research did not find these associations. Similarly, there is debate regarding the relationship between family economic status and IA. An Egyptian study suggests that students from better economic conditions have a higher risk of IA and are more likely to exhibit depressive symptoms (10), while another study from Hong Kong argues that financial hardship is a significant risk factor for adolescent IA (75). However, Our study found no significant correlation between family economic status and IA.

The inconsistencies in these findings may be due to differences in the study populations. Our research focused on medical students with a psychiatric major, who generally have better mental health, which may reduce the influence of certain social factors on IA. Additionally, cultural differences across countries may contribute to these discrepancies. Further research is needed to explore these differences between medical students with a psychiatric major and other populations.

Overall, our findings have important practical implications. Medical students with a psychiatric major demonstrated better mental health and a lower prevalence of IA compared to general medical students, highlighting the potential benefits of incorporating psychology- or psychiatry-related coursework into medical education. Enhancing students’ mental health literacy may contribute to the prevention and management of mental disorders, ultimately improving the overall well-being of the student population. From a clinical perspective, our results also suggest that psychiatrists could benefit from providing psycho-education to patients. Improving patients’ mental health literacy may facilitate better symptom understanding and potentially lead to symptom reduction. In addition, our study revealed a strong association between campus bullying and IA. This suggests that when assessing and treating individuals with IA, it may be helpful to inquire about their bullying history. Strengthening support systems within schools and families for those affected could be a valuable component of prevention and intervention efforts.

Specifically, based on the findings of this study, we recommend systematically incorporating content on Internet addiction into undergraduate mental health education and psychiatric curricula. This should include education on its etiological mechanisms, clinical manifestations, adverse consequences, prevention strategies, and treatment approaches. For example, classroom instruction could place greater emphasis on cognitive behavioral therapy (CBT), along with practical training, to enhance students’ competence in applying evidence-based interventions.

In addition to curriculum development, routine assessment of students’ mental health should be strengthened. Individualized interventions should be offered for students experiencing depression, anxiety, or Internet addiction. Furthermore, schools should collaborate with families and broader social support systems to establish comprehensive mental health interventions, while also identifying and addressing potential psychological risk factors such as campus bullying.

## 5 Limitations

First, the study sample was drawn from a single university in China, which limits the generalizability of the findings to other regions or academic disciplines. Second, due to the cross-sectional nature of the study, causal relationships cannot be established. Third, the reliance on self-reported data may introduce response biases, such as underreporting of IA or other sensitive behaviors. Finally, the findings may be influenced by specific cultural or educational factors within Chinese medical education. Notably, as undergraduate psychiatric majors are uncommon outside China, the findings may not generalize to other medical education systems. Therefore, the findings should be interpreted with caution when considering generalizability across different educational contexts.

To address the limitations of the present study, future research should consider recruiting participants from multiple universities or regions, both within and outside China, to improve the generalizability of findings across different educational and cultural contexts. Longitudinal studies are also needed to clarify the causal relationships between academic progression, psychological coursework, mental health status, and internet addiction. Additionally, future research could examine whether psychology or psychiatry-related coursework serves as a protective or preventive factor against mental health problems and IA among general medical students. These findings could inform the design of curriculum-based interventions aimed at promoting psychological resilience in medical education.

## 6 Conclusion

This study highlights the protective role of psychiatry-specific education in mitigating IA and associated mental health issues. First, the study found that medical students with a psychiatric major appeared to experience fewer symptoms of IA, depression, and anxiety compared to medical students reported in previous literature. Second, higher academic years were associated with reduced IA, through partial mediation by decreased impulsivity and depression. This may be attributed to the increasing psychological literacy gained from coursework in the field. Finally, poor peer relationships, poor relationships with parents, and experiences of peer bullying were significantly correlated with IA, highlighting the need for targeted social support interventions to address IA. These findings suggest that enhancing psychological education and providing social support could be valuable strategies in mitigating IA.

## Data Availability

The raw data supporting the conclusions of this article will be made available by the authors, without undue reservation.
